# Phloretin attenuates hyperuricemia‐induced endothelial dysfunction through co‐inhibiting inflammation and GLUT9‐mediated uric acid uptake

**DOI:** 10.1111/jcmm.13176

**Published:** 2017-04-12

**Authors:** Shuyun Liu, Yujia Yuan, Yijie Zhou, Meng Zhao, Younan Chen, Jingqiu Cheng, Yanrong Lu, Jingping Liu

**Affiliations:** ^1^ Key Laboratory of Transplant Engineering and Immunology NHFPC; Regenerative Medicine Research Center West China Hospital Sichuan University Chengdu China

**Keywords:** uric acid, phloretin, GLUT9, Inflammation, endothelial dysfunction

## Abstract

Hyperuricemia is an important risk factor for cardiovascular and renal diseases. Phloretin had shown antioxidant and anti‐inflammatory properties, but its role in endothelial injury is rarely reported. In this study, we aimed to investigate the protective effect of phloretin on UA‐induced injury in human umbilical vein endothelial cells. The effects of UA and phloretin on cell viability, inflammation, THP‐1 monocyte adhesion, endothelial cell tube formation, GLUT9 expression and UA uptake in human umbilical vein endothelial cells were evaluated. The changes of nuclear factor‐kappa B/extracellular regulated protein kinases signalling were also analysed. Our results showed that UA reduced cell viability and tube formation, and increased inflammation and monocytes adhesion in human umbilical vein endothelial cells in a dose‐dependent manner. In contrast, phloretin significantly attenuated pro‐inflammatory factors expression and endothelial injury induced by UA. Phloretin inhibited the activation of extracellular regulated protein kinases/nuclear factor‐kappa B pathway, and reduced GLUT9 and it mediated UA uptake in human umbilical vein endothelial cells. These results indicated that phloretin attenuated UA‐induced endothelial injury *via* a synergic mechanism including direct anti‐inflammatory effect and lowering cellular UA uptake. Our study suggested that phloretin might be a promising therapy for hyperuricemia‐related cardiovascular diseases.

## Introduction

Uric acid (UA) is the end product of purine degradation in human and non‐human primate, while it is further converted to allantoin by enzyme uricase in other mammals. Although the normal level of UA may provide antioxidant defence in the human body, abnormal high level of blood UA (hyperuricemia) is often associated with gout, metabolic syndrome, insulin resistance, hypertension, T2DM and chronic renal diseases (CKD) 1, 2, 3, 4. Epidemiological evidences suggest that hyperuricemia and cardiovascular diseases are highly related 3, 4. Endothelial dysfunction is an initial and essential factor in the progression of cardiovascular diseases 5, and UA could directly induce endothelial cells injury *via* multiple mechanisms such as oxidative stress and inflammation 6. Therefore, development of novel therapy to attenuate UA‐induced vascular endothelial injury is needed.

Increased evidences showed that some natural products such as polyphenols had beneficial effects on cardiovascular health. Phloretin belongs to the chalcone class of flavonoid compounds that abundance in apple and pear trees, which had shown various biological properties including regulation of glucose transporters, anticancer, antioxidant and anti‐inflammatory activities 7, 8, 9, 10, 11 Previous studies reported that phloretin reduced the expression of pro‐inflammatory cytokines in epithelial cells and LPS‐stimulated macrophages 8, and reduced the activation of human platelets 9. Phloretin also was found to induce apoptosis in cancer cells and promote lipolysis in adipocytes 10, 11. However, the effect of phloretin on UA‐induced vascular endothelial injury has not been determined.

Cellular UA uptake is a complex processes with balance between UA reabsorption and excretion 12, 13. As UA is an organic anion, the transport of UA from extracellular to cytoplasm needs membrane transporters. Several UA transporter proteins such as URAT1 (*SLC22A12*), GLUT9 (*SLC2A9*), *ABCG2*, OATs and NPTs had been previously reported 12, 13, 14, 15, and mutation of *SLC2A9*,* SLC22A12* and *ABCG2* had been proved could affect renal UA excretion and blood UA levels 14, 15. It had been reported that UA uptake also affected the degree of inflammation, and block of UA uptake by probenecid reduced the UA‐induced inflammation in pancreatic β cells 16. Previous study reported that phloretin inhibited UA uptake in GLUT9 over‐expressed *X. laevis oocytes* 17. Based on these reports, we concluded that phloretin may be beneficial for reducing UA‐induced endothelial injury through its potency to regulate inflammation and UA uptake.

In this study, we aimed to study the protective role of phloretin on UA‐induced endothelial injury in HUVECs. We evaluated the effects of phloretin on GLUT9 expression, UA uptake and endothelial cell injury in HUVECs, and explored the potential protective mechanism of phloretin on UA‐induced endothelial injury.

## Materials and Methods

### Cell culture and treatment

HUVECs obtained from ScienCell Research Laboratories (USA) were cultured in supplemented EBM‐2 basal media (EndoGRO™VEGF Supplement kit. Millipore). Uric acid (Sigma, Saint Louis, USA) was dissolved in warmed serum‐free EBM‐2 medium and filtered with 0.22 μm syringe filter unit (Millipore) as previously described 18. UA crystals were not detected during the cell experiments by polarizing microscopy. Cells were pre‐treated with phloretin for 30 min. before addition of UA.

### Small interfering RNA (siRNA) transfection

*SLC2A9* (GLUT9) and control siRNAs were purchased from Shanghai Genechem Co., Ltd. (Shanghai, China), and their sequences were listed in Table S1. The siRNA transfection was performed using Lipofectamine 2000 (Life) according to the manufacturer's protocol. Briefly, diluted siRNA solution was mixed with Lipofectamine 2000 and incubated at room temperature for 30 min., and then, the work solution of siRNA was added to cells. The culture medium was changed at 6 hrs after transfection, and the GLUT9 protein expression in HUVECs was measured at 24 hrs after transfection.

### Uric acid uptake assay

HUVECs were washed with PBS twice before experiment, and then UA uptake assay was performed in serum‐free EBM‐2 media containing 500 μM UA. The culture supernatant was collected at 30 and 60 min. after incubation, and the concentration of UA was measured by commercial uric acid assay kit (Sigma, USA). After experiment, cells were digested, collected and counted using haemocytometer. The UA uptake rate was determined by normalizing the UA level to cell number in each sample.

### Cell viability assay

HUVECs were seeded in 96‐well plate and treated with indicated conditions. After treatment, 10 μl of CCK‐8 solution (Dojindo, Kumamoto, Japan) was added to each well, and the plates were incubated for 2 hrs at 37°C. The absorbance at 450 nm was measured by microplate reader (BioTek Instruments Inc, USA). The cell viability was calculated by normalizing optical density of the experimental group to the control group.

### Quantitative real‐time PCR

Total RNA was extracted using Trizol reagent (Gibco, Life Technologies, CA, USA) and reverse‐transcribed into cDNA using an iScript cDNA synthesis kit (Bio‐Rad, USA). Real‐time polymerase chain reaction (Real‐time PCR) was performed on CFX96 real‐time PCR detection system (Bio‐Rad, USA) with SYBR Green (Bio‐Rad, USA). Primers used in this study were listed in Table S2. The data were analysed using Bio‐Rad CFX Manager software, and the relative change of mRNA expression was calculated by delta‐delta Ct method with β‐actin as reference gene.

### Western blot

Cells were lysed in radioimmuno‐precipitation assay (RIPA) buffer, supplemented with protease inhibitors (Calbiochem, CA, USA) and phosphatase inhibitors (Calbiochem). The protein concentration was determined by BCA Protein Assay Kit (CWBIO, China). Equal amount of protein was subjected to electrophoresis on 10% sodium dodecyl sulphate–polyacrylamide gel (SDS‐PAGE), and then transferred to polyvinylidene difluoride membranes (PVDF, Merck Millipore). The membranes were blocked with 5% non‐fat milk and incubated with primary antibodies against p‐NFκB (1:1000, Abcam, MA, USA) and NFκB (1:500, Santa Cruz Biotechnology, CA, USA), GLUT9 (1:1000, Abcam), IL‐1β (1:500, ABclonal Biotechnology), OAT1 (1:1000, ABclonal ), URAT1 (1:1000,ABclonal) ICAM‐1 (1:1000, Cell Signaling Technology, MA, USA), MCP‐1 (1:1000, Abcam), VCAM‐1 (1:500, Proteintech, USA) overnight at 4°C. After washed with PBST, the PVDF membrane was incubated with horseradish peroxidase‐conjugated secondary antibody (1:2000, Santa Cruz) at 37°C for 1 hr. An enhanced chemiluminescence kit (Millipore) was used for signal detection. Protein bands were quantified by NIH Image J software and normalized to the expression of β‐actin.

### Monocyte adhesion assay

Human monocyte cell line THP‐1 was cultured in RPMI 1640 supplemented with 10% FBS (Gibco) and cultured at 37°C in humidified 5% CO_2_ incubator. The THP‐1 cells were pre‐labelled with CM‐DiI (2 μM, Molecular Probes, Thermo Fisher Scientific) for 5 min. at 37°C and then for an additional 15 min. at 4°C in RPMI 1640. After treatment, the THP‐1 cells were added to each well of HUVECs and co‐cultured for 2 hrs. Each well was washed three times with PBS, and then, images were randomly captured at ten regions in each well at 200 × magnification using fluorescence microscope (IX71, Olympus, Japan).

### Endothelial cell tube formation assay

Matrigel matrix (BD Biosciences, CA, USA) was thawed at 4°C, then pipetted into pre‐chilled 96‐well plates (50 μl matrigel/well) and incubated at 37°C for 1 hr. After treatment, HUVECs were collected and placed on the layer of matrigel (2× 10^4^ cells/well). After 2 hr of incubation at 37°C, the tube‐like structure of endothelial cells was examined by inverted microscope (Eclipse TS100, Nikon, Japan) at 100 × magnifications. Branching points in ten random fields per well was quantified by Image‐Pro Plus software. Inhibition percentage was expressed as percentage of the control (100%). The assay was repeated three times independently.

### Immunofluorescence (IF) staining

Cells were fixed with 4% paraformaldehyde in PBS for 10 min. at room temperature and then washed twice with PBS and permeabilized with 0.3% Triton X‐100 for 10 min. After blocking in 1% BSA for 60 min., the slides were incubated with mouse anti‐NFκB antibody (1:200), mouse anti‐ICAM‐1 antibody (1:200), rabbit anti‐MCP‐1 antibody (1:200) overnight at 4°C followed by incubation with fluorescein isothiocyanate (FITC)‐conjugated or tetramethylrhodamine (TRITC)‐conjugated secondary antibody for 1 hr at 37°C. Nuclei were visualized by staining with DAPI. Digital images were captured by fluorescent microscope.

### Statistical analysis

All data were presented as mean ± S.D., and analysed using one‐way anova test with a value of *P* < 0.05 considered to significant difference. Comparison between two groups of values was performed by *t*‐test.

## Results

### UA‐induced HUVECs injury and inflammation in a dose‐dependent manner

**Figure 1:**
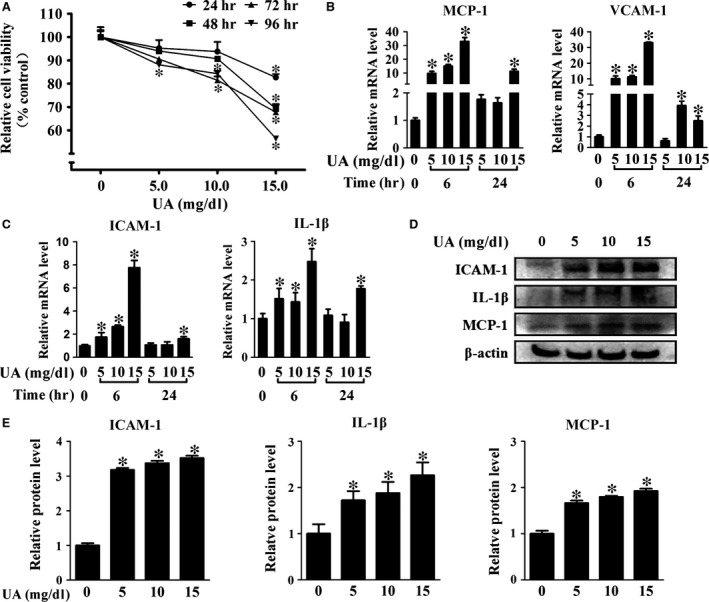
Effects of UA on cell viability and inflammation in HUVECs. (**A**) Cell viability of HUVECs assayed by CCK8. (**B‐C**) Real‐time PCR analysis of *MCP‐1*,*VCAM‐1*,*ICAM‐1* and *IL‐1*β mRNA. (**D**) Western blot of ICAM‐1, IL‐1β and MCP‐1 protein, cell treated with UA for 24h. (**E**) Quantification analysis of protein level related to control (*P < 0.05, UA group *versus* control). HUVECs: human umbilical vein endothelial cells, NFκB/ERK: nuclear factor‐kappa B/extracellular regulated protein kinases.

**Figure 2:**
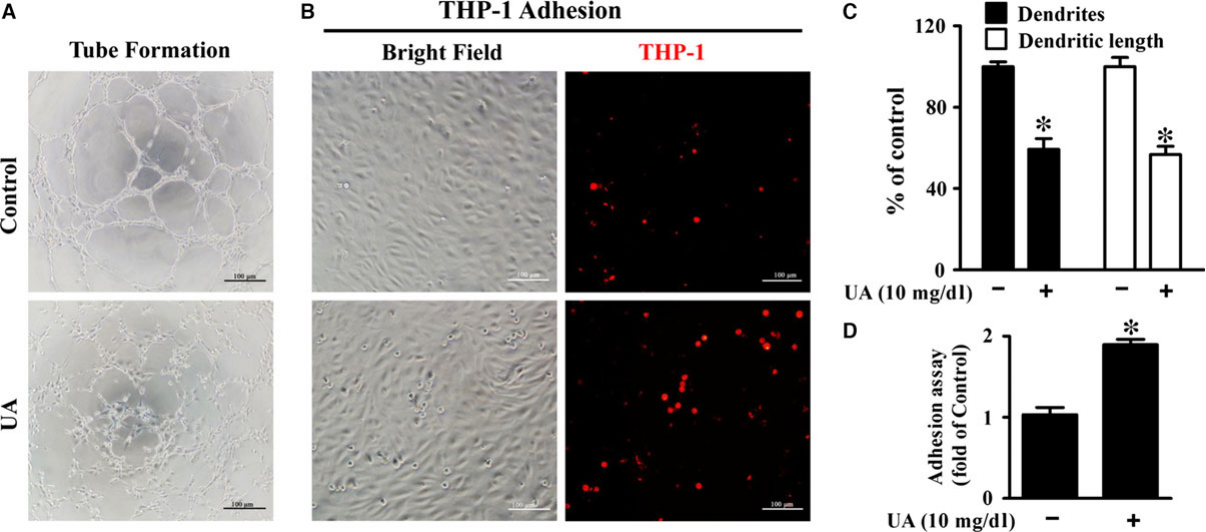
Effect of UA on endothelial dysfunction in HUVECs. (**A**) Representative micrographs of tube formation assay, cell treated with UA for 24h (scale bar = 100 μm). (**B**) CM‐Dil‐labelled THP‐1 cell (red) adhesion to HUVECs, and cell treated with UA for 24h (scale bar = 100 μm). (**C**) Quantification results of tube formation assay. (**D**) Quantification results of THP‐1 adhesion to HUVECs (*P < 0.05, UA group *versus* control). HUVECs: human umbilical vein endothelial cells, NFκB/ERK: nuclear factor‐kappa B/extracellular regulated protein kinases.

As shown in Figure 1A, UA significantly reduced the cell viability of HUVECs in a dose‐ and time‐dependent manner. UA also promoted the expression of pro‐inflammatory factors including MCP‐1, VCAM‐1, ICAM‐1 and IL‐1β in HUVECs in a dose‐dependent manner (Fig. 1B‐E). High level of UA inhibited tube formation including the dendrites and dendritic length (Fig. 2A and C), and enhanced THP‐1 monocytes adhesion in HUVECs significantly (Fig. 2A and D).

### Phloretin attenuated UA‐induced inflammation and endothelial dysfunction

**Figure 3:**
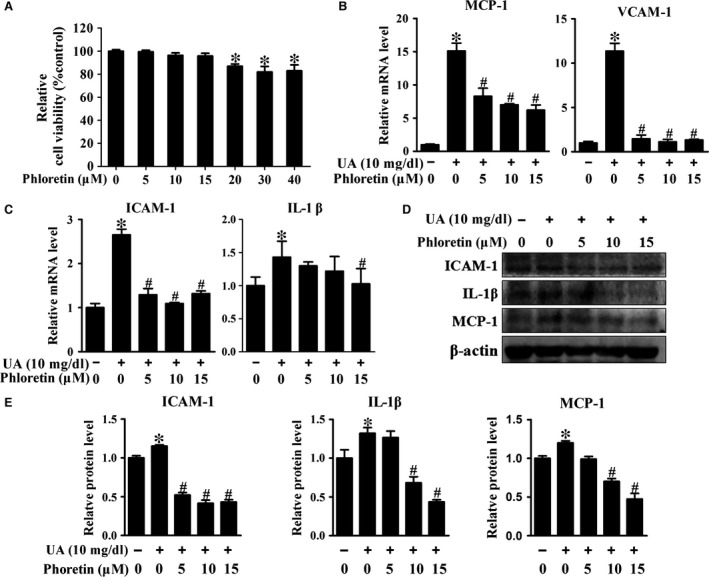
Effect of phloretin on UA‐induced inflammation in HUVECs. (**A**) Cell viability of HUVECs assayed by CCK8, cell treated with phloretin for 24h. (**B‐C**) Real‐time PCR analysis of *MCP‐1*,*VCAM‐1*,*ICAM‐1* and *IL‐1*β mRNA, and cell treated with UA with or without phloretin for 24h. (**D**) Western blot and (**E**) quantification analysis of ICAM‐1, IL‐1β and MCP‐1 protein (*P < 0.05, UA group *versus* control, ^#^ *P* < 0.05, phloretin group *versus* UA group). HUVECs: human umbilical vein endothelial cells, NFκB/ERK: nuclear factor‐kappa B/extracellular regulated protein kinases.

**Figure 4:**
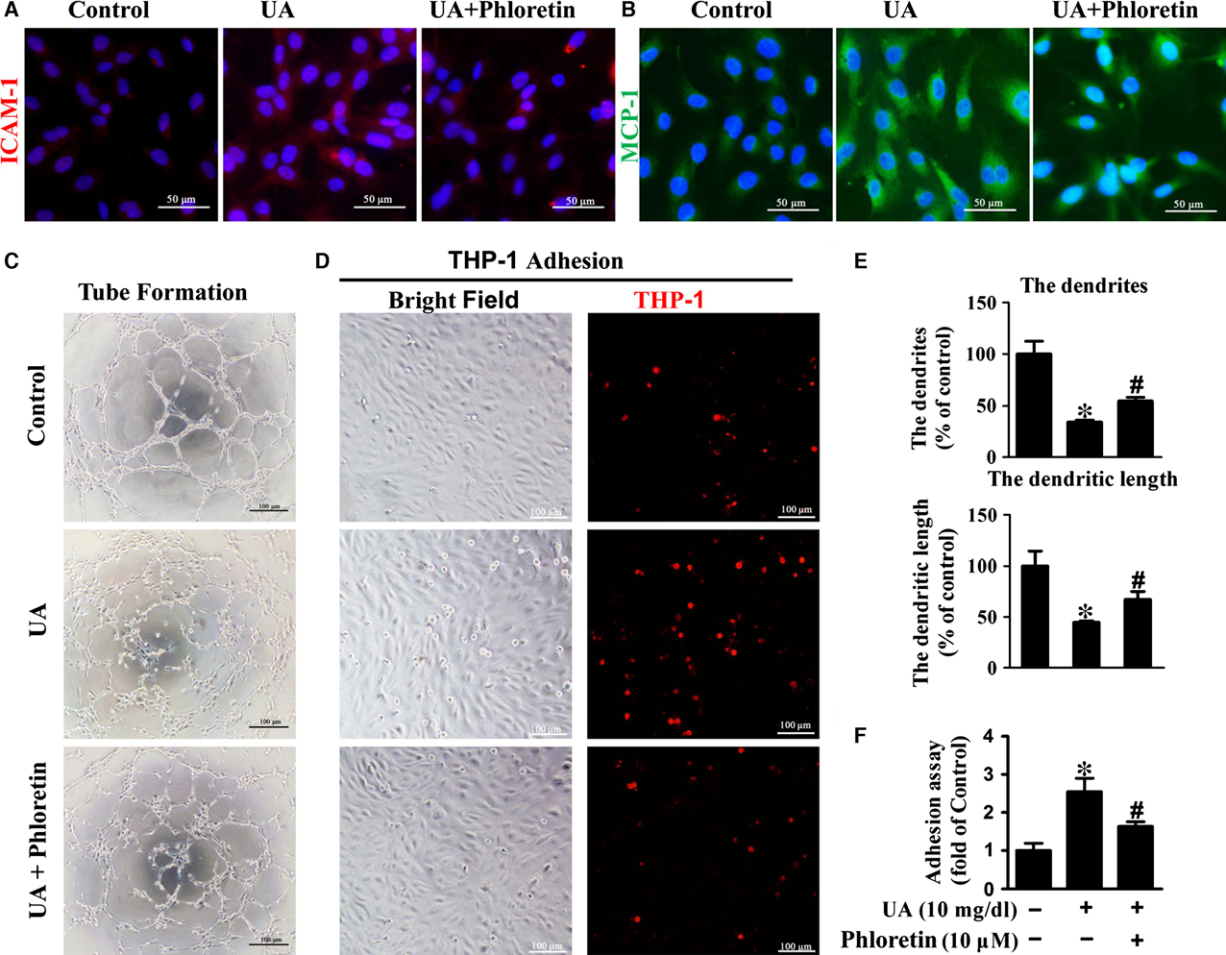
Effect of phloretin on UA‐induced endothelial dysfunction in HUVECs. (**A‐B**) Immunofluorescent staining of ICAM‐1 (red) and MCP‐1(green) in HUVECs (scale Bar = 50 μm). (**C**) Representative micrographs of tube formation assay (scale bar = 100 μm). (**D**) THP‐1 cell (red) adhesion to HUVECs assay (scale bar = 100 μm). (**E**) Quantification of tube formation assay. (**F**) Quantification results of THP‐1 adhesion assay (*P < 0.05, UA or phloretin group *versus* control, ^#^ *P* < 0.05, phloretin group *versus* UA group). HUVECs: human umbilical vein endothelial cells, NFκB/ERK: nuclear factor‐kappa B/extracellular regulated protein kinases.

Because high dose of phloretin (>20 μM) induced obvious cytotoxicity in HUVECs (Fig. 3A), relative lower dose of phloretin (5‐15 μM) was used in the following experiments. Our results showed that phloretin significantly decreased UA‐induced pro‐inflammatory factors expression including ICAM‐1, MCP‐1 and IL‐1β (Fig. 3B‐E). IF staining results also showed that phloretin reduced the expression of ICAM‐1 and MCP‐1 that induced by UA (Fig. 4A‐B). Furthermore, phloretin improved the endothelial tube formation and decreased monocytes adhesion of HUVECs in UA‐treated HUVECs (Fig. 4C‐F).

### Phloretin had weakly direct anti‐inflammatory effect at relative lower does

**Figure 5:**
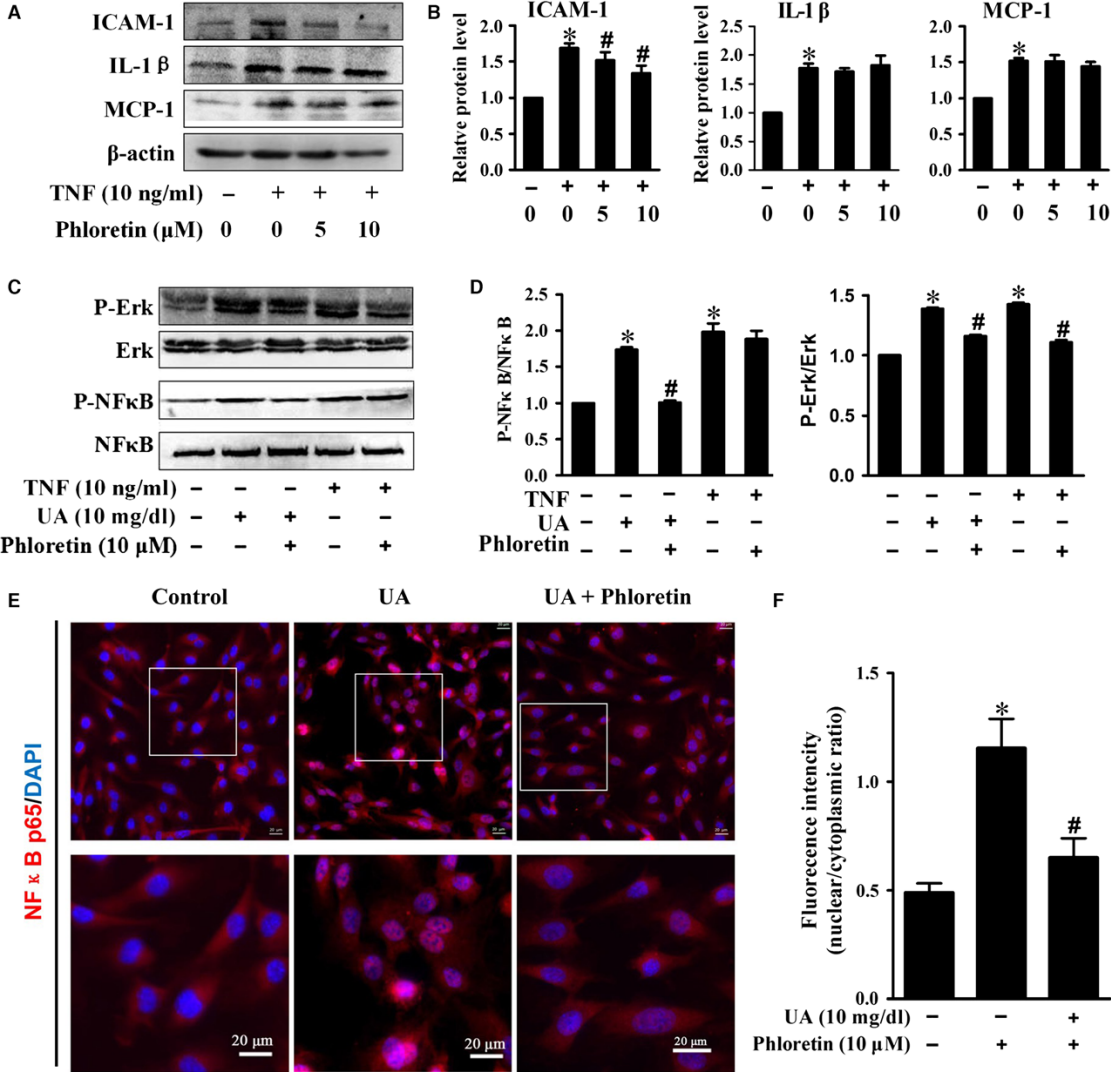
Effect of phloretin on UA/TNF‐α‐induced inflammation and NFκB/ERK activation. (**A‐B**) Western blot and quantification analysis of ICAM‐1, IL‐1β and MCP‐1 protein, cell treated with TNF‐α and phloretin. (**C‐D**) Western blot and quantification analysis of p‐ERK/ERK and p‐NFκB p65/NFκB p65 levels. (**E**) Immunofluorescent staining and (**F**) quantification results of NFκB p65 nuclear translocation in HUVECs (scale bar = 20 μm) (*P < 0.05, UA or TNF group *versus* control, ^#^ *P* < 0.05, phloretin group *versus* UA or TNF group). HUVECs: human umbilical vein endothelial cells, NFκB/ERK: nuclear factor‐kappa B/extracellular regulated protein kinases.

In TNF‐α treated HUVECs, phloretin can only reduced the ICAM‐1 level, but had no influence on IL‐1β and MCP‐1 expression (Fig. 5A‐B). Both UA and TNF‐α increased p‐ERK and p‐NFκB expression in HUVECs, and phloretin significantly reduced p‐NFκB/p‐ERK level and nuclear translocation of NF‐κB p65 induced by UA, but it only decreased p‐ERK level induced by TNF‐α (Fig. 5C‐F).

### Phloretin inhibited UA‐induced inflammation *via* lowering GLUT9‐mediated UA uptake

**Figure 6:**
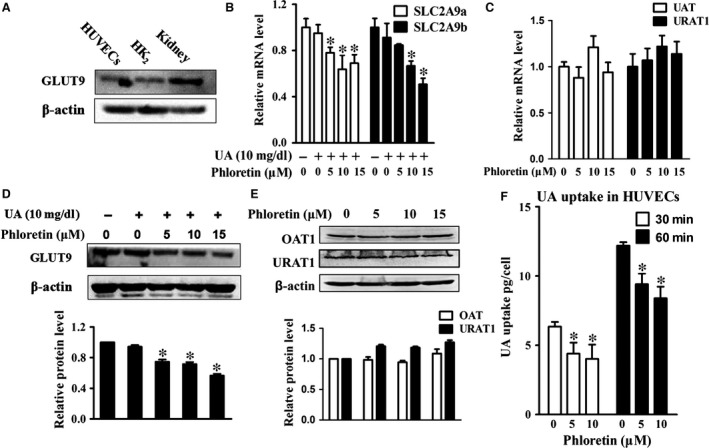
Effect of phloretin on GLUT9 expression and UA uptake in HUVECs. (**A**) GLUT9 expression in HUVECs, HK‐2 cells and rat kidney tissue. (**B‐C**) Real‐time PCR analysis of *SLC2A9*,*UAT* and *URAT1* mRNA. (**D‐E**) Western blot and quantitative analysis of GLUT9, OAT and URAT1 protein. (**F**) Effect of phloretin on UA uptake in HUVECs (*P < 0.05, UA with phloretin group *versus* control). HUVECs: human umbilical vein endothelial cells, NFκB/ERK: nuclear factor‐kappa B/extracellular regulated protein kinases.

**Figure 7:**
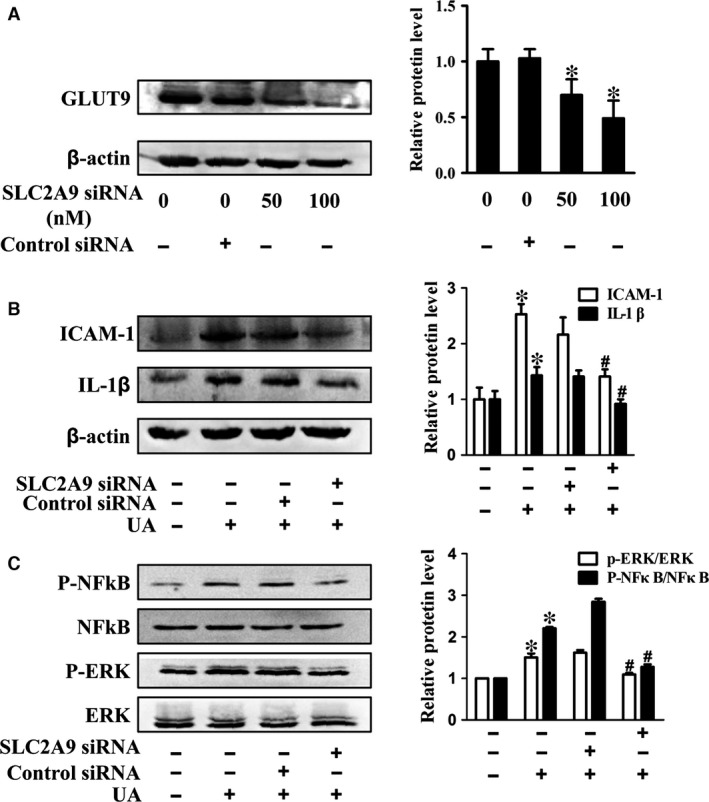
Effect of GLUT9 inhibition on UA uptake and inflammatory response in HUVECs. (**A**) Western blot analysis of GLUT9 expression in siRNA treated HUVECs (24 hrs). (**B‐C**) Western blot analysis of *SLC2A9* siRNA on ICAM‐1, IL‐1β and NFκB/ERK activation in UA‐treated HUVECs (*P < 0.05, UA group *versus* control, ^#^ *P* < 0.05, siRNA group *versus* UA group). HUVECs: human umbilical vein endothelial cells, NFκB/ERK: nuclear factor‐kappa B/extracellular regulated protein kinases.

Our results showed that GLUT9 expressed in HUVECs, which was consistent with the positive controls including HK2 cell and rat kidney (Fig. 6A). Phloretin significantly reduced GLUT9 expression (Fig. 6B‐D), but had no influence on other UA transporters including URAT1 and OAT1 (Fig. 6C‐E). In addition, phloretin significantly lowered UA uptake in HUVECs (Fig. 6F). Furthermore, knockdown of GLUT9 by siRNA significantly inhibited IL‐1β and ICAM‐1 expression (Fig. 7A‐B), as well as reduced p‐NFκB and p‐ERK levels in UA‐treated HUVECs (Fig. 7C).

## Discussion

It was well documented that there was a strong relationship between hyperuricemia and cardiovascular diseases, and high level of UA‐induced endothelial injury *via* multiple mechanisms such as oxidative stress, growth inhibition, epithelial mesenchymal transition (EMT) and inflammation 19, 20, 21, 22. However, the potential role of phloretin in UA‐induced endothelial injury in vascular endothelial cells is not clear.

Extensive epidemiologic studies suggest that elevated blood UA level is highly associated with diabetes, cardiovascular and renal diseases 3, 23, and increasing evidences indicate that UA‐induced endothelial dysfunction is a fundamental mechanism 5. It had been reported that UA aggravated endothelial dysfunction and raised the risk of diabetes in hypertensive patients 23. However, the link between UA transport and endothelial injury is still incompletely understood. In this study, we proved that UA reduced HUVECs viability in a time‐ and dose‐dependent manner, which was similar to previous reports in pancreatic β cell and renal HK‐2 cell 16, 24. Our results further showed UA induced the release of chemokines and adhesion molecules, disturbed endothelial cell tube formation and increased monocyte‐endothelial cell interaction in HUVECs in a dose‐dependent manner. These results suggested that cellular UA level mediated by UA transporter might affect the degree of inflammation and endothelial dysfunction in HUVECs directly.

Inflammation plays a key role in the initiation and progression of vascular injury response to stress stimulation, and the release of inflammatory factors further aggravated endothelial injury *via* directly recruiting inflammatory cells such as neutrophils, monocytes and macrophages 25. NF‐κB and ERK are major pathways in mediating immune and inflammatory response, which had been involved in UA‐induced inflammatory factors expression 26, 27. Our data showed that high level of UA‐induced inflammation and endothelial injury *via* activating NF‐κB/ERK pathway. Phloretin is a natural polyphenolic compound derived from rosaceae family plants, which had shown antioxidant and anti‐inflammatory properties 7, 8, 9. In this study, we observed that high level of phloretin (>20 μM) had obvious cytotoxicity on HUVECs, while relative lower level of phloretin (5‐15 μM) did not affect cell viability. In addition to UA, we also assayed the direct effect of phloretin on inflammation in TNF‐α treated HUVECs, because TNF‐α is a well‐known pro‐inflammatory cytokine. Our results showed that phloretin significantly eliminated UA‐induced IL‐1β, ICAM‐1 and MCP‐1 expression and NF‐κB/ERK activation, but it only decreased TNF‐α‐induced ICAM‐1 and p‐ERK expression, and did not affect MCP‐1, IL‐1β and p‐NF‐κB levels in HUVECs. These results suggested that phloretin had weakly direct anti‐inflammatory effect at relative low dose, thus other mechanisms might be involved the protective effect of phloretin on UA‐induced HUVECs injury.

Although the anti‐inflammatory effect of the phloretin has been reported, few studies investigated the role of phloretin on cellular UA uptake and UA transporters. UA transporters such as OATs, URAT1 and GLUT9 played a key role in maintaining UA homoeostasis in various types of cells and tissues 12, 13. GLUT9 (*SLC2A9*) had been recognized as a high capacity UA transporter in human and rodents 17, 28, and plays an important role in mediating blood UA level 14, 15, 29. GLUT9 mutation caused renal hypouricemia by reducing UA reabsorption in the renal proximal tubules 29, 30. GLUT9 expressed broadly in various tissues such as liver, lung, heart, kidney, intestine, leucocytes and chondrocytes 12, 13. It had been shown that blocking of UA uptake inhibited the activation of ERK induced by UA in pancreatic β cell 16, which suggested that blocking UA transport into cells may be a promising strategy to ameliorate UA‐induced inflammation and endothelial injury. Phloretin had shown potential inhibit effect on UA transport, which reduced about 50% of UA uptake in *xenopus laevis* oocyte model at a dose of 1 mM 17, but its effect mechanism was not clear. In this study, we observed that phloretin effectively inhibited UA uptake in HUVECs in a dose‐dependent manner. Phloretin also significantly reduced GLUT9 expression, but did not affect other UA transporters such as OAT1 and UART1 expression in HUVECs. Previous study found that GLUT9 had high similarity in sequence to the glucose transporter (GLUT) family 9, while phloretin is a common inhibitor of GLUT family. Although the specific mechanism is unclear, our results suggested that phloretin reduced UA uptake mainly *via* inhibiting GLUT9‐mediated UA transport in HUVECs. Furthermore, we used siRNA to knockdown GLUT9 and assayed the effect of GLUT9 inhibition on inflammation in HUVECs. Our results showed that GLUT9 siRNA significantly decreased inflammatory factors expression and NF‐κB/ERK activation induced by UA, which was similar to the effect of phloretin on UA‐treated HUVECs. These results suggested that inhibition of GLUT9‐mediated UA transport also contributed to the protective effect of phloretin on UA‐induced endothelial injury.

Taken together, hyperuricemia is strongly associated with endothelial dysfunction and development of diabetes, cardiovascular and renal diseases. Phloretin significantly attenuates UA‐induced vascular injury due to its anti‐inflammatory and lowering UA action. In addition, phloretin is a natural compound abundant in plant trees, and its therapeutic efficacy can be further improved by chemical or biological modification. Therefore, phloretin provides a novel and cost‐effective approach to prevent hyperuricemia‐associated vascular complications.

## Conclusions

In conclusion, our study demonstrated that high level of UA‐induced endothelial dysfunction *via* activation of inflammatory signalling in HUVECs, whereas phloretin significantly attenuated inflammation and endothelial injury induced by UA. We further revealed that phloretin decreased UA‐induced HUVECs injury *via* a synergic mechanism including direct anti‐inflammatory effect and blocking of GLUT9‐mediated UA uptake. This study suggested that phloretin might be a promising therapy to hyperuricemia‐related cardiovascular diseases.

## Conflict of interests

The authors confirm that there are no conflict of interests.

## Supporting information


**Table S1** The sense and antisense sequences for siRNA in this study.
**Table S2** Primers sequences for real‐time PCR in this study.
