# Supplementary material for: Phloretin attenuates hyperuricemia‐induced endothelial dysfunction through co‐inhibiting inflammation and GLUT9‐mediated uric acid uptake
Source: J Cell Mol Med. 2017 Apr 12;21(10):2553–62. doi: 10.1111/jcmm.13176 (PMC5618667; doi:10.1111/jcmm.13176)
Supplement: Supplementary file 1 — Table S1 The sense and antisense sequences for siRNA in this study. Table S2 Primers sequences for real‐time PCR in this study. [file JCMM-21-2553-s001.doc]

**Supplementary Material**

Table S1. The sense and antisense sequences for siRNA in this study

| siRNA | sequences |
| --- | --- |
| SLC2A9 | sense: 5'-GCAAUUUGGUUCUAUACCATT-3' |
|  | antisense: 5'-UGGUAUAGAACCAAAUUGCTT-3' |
| Control | sense: 5'-UUCUCCGAACGUGUCACGUTT-3' |
|  | antisense: 5'-ACGUGACACGUUCGGAGAATT-3 |

Table S2. Primers sequences for real-time PCR in this study

| Gene | sequences |
| --- | --- |
| β-actin | F: 5'-CCACGAAACTACCTTCAACTCC-3' |
|  | R: 5'-GTGATCTCCTTCTGCATCCTGT-3' |
| SLC2A9a | F: 5'-TTGCTCTGGTCTGTGACTGTG-3' |
|  | R: 5'-TTGCTCTGGTCTGTGACTGTG-3' |
| SLC2A9b | F: 5'-GACTCCAGAGGGGCATGAAAA-3' |
|  | R: 5'-AGCAGGACCAGTCCAATTTCT-3' |
| IL-1β | F: 5'-TGGCAGAAAGGGAACAGAAA-3' |
|  | R: 5'-CTGGCTGATGGACAGGAGAT-3' |
| MCP-1 | F: 5'-CAGCCAGATGCAATCAATGCC-3' |
|  | R: 5'-TGGAATCCTGAACCCACTTCT-3' |
| ICAM-1 | F: 5'-ACCATCACCGTGTATTCGTT-3' |
|  | R: 5'-GCTGGCGGCTCAGTATCT-3' |
| VCAM-1 | F: 5'-CCCCCAGAGATACAACCGTC-3' |
|  | R:5'-CTGCCTGCTCCACAGGATTT-3' |
